# Prostaglandin I2 enhances cough reflex sensitivity to capsaicin in the asthmatic airway

**DOI:** 10.1186/1745-9974-3-2

**Published:** 2007-01-12

**Authors:** Yoshihisa Ishiura, Masaki Fujimura, Kouichi Nobata, Yoshitaka Oribe, Miki Abo, Shigeharu Myou

**Affiliations:** 1 The Department of Internal Medicine, Toyama City Hospital, Toyama, Japan; 2 Respiratory Medicine, Cellular Transplantation Biology, Kanazawa University Graduate School of Medicine, Kanazawa, Japan

## Abstract

Inflammatory mediators are involved in the pathogenesis of airway inflammation, but the role of prostaglandin I2 (PGI2) remains obscure. This study was designed to investigate the role of PGI2 in cough reflex sensitivity of the asthmatic airway, which is characterized by chronic eosinophilic airway inflammation. The effect of beraprost, a chemically and biologically stable analogue of PGI2, on cough response to inhaled capsaicin was examined in 21 patients with stable asthma in a randomized, placebo-controlled cross over study. Capsaicin cough threshold, defined as the lowest concentration of capsaicin eliciting five or more coughs, was measured as an index of airway cough reflex sensitivity. The cough threshold was significantly (p < 0.05) decreased after two weeks of treatment with beraprost [17.8 (GSEM 1.20) μM] compared with placebo [30.3 (GSEM 1.21) μM]. PGI2 increases cough reflex sensitivity of the asthmatic airway, suggesting that inhibition of PGI2 may be a novel therapeutic option for patients with asthma, especially cough predominant asthma.

## Background

Chronic cough is one of the commonest respiratory symptoms. Cough has been considered to be a defense mechanism of the airway to remove irritant particles or excess mucus, whereas non-productive cough, which is not associated with the clearance of the tracheobronchial mucus, may occur via increased cough reflex sensitivity. Inflammatory mediators such as prostaglandins may adjust the cough reflex sensitivity. However, little is known about how cough reflex sensitivity is influenced by airway inflammatory processes. Although our previous study has clearly shown that arachidonate cyclooxygenase products can modulate airway cough reflex sensitivity to inhaled capsaicin [1], the effects of other mediators remains unknown.

It has been recognized that prostaglandin I2 (PGI2, prostacyclin) is the most abundant prostanoid generated on IgE-dependent challenge of human lung tissue in vitro [2,3]. Others reported that alveolar macrophages are able to synthesize large amount of PGI2 [4]. These findings indicate that PGI2 may play some role in the asthmatic airway and can affect airway cough reflex sensitivity. This study was conducted to elucidate this hypothesis. We investigated the effect of oral administration of beraprost, a chemically and biologically stable analog of PGI2 {sodium (±)-4[(1R, 2R, 3aS, 8bS)-1, 2, 3a, 8b-tetrahydro-2-hydroxyl 2[(3S, 4RS)-3-hydroxy-4-methyl-oct-6-yne-(E)-l-enyl]-5-cyclopenta [b] benzofuranyl] butyrate}, on cough reflex sensitivity to inhaled capsaicin in patients with stable asthma [5].

## Subjects and Methods

### Subjects

Twenty-one patients with bronchial asthma (12 males and 9 females) with a mean age of 73.2 ± 1.5 (± SEM) (range 54–83) yrs participated in this study. All patients were lifetime nonsmokers or ex-smokers with no history of viral infection for at least 4 weeks prior to the study. Characteristics of individual patients are shown in Table 1. Informed consent was obtained from all subjects. This study was approved by the Ethics Committee of our hospital.

**Table 1:** Clinical characteristics of patients

Patient number	Age (yr)	Sex	Height (cm)	Type	Severity	Total IgE in serum (IU/ml)	Specific IgE in serum	Complication of allergic disease	PC20-FEV1 (mg/ml)*	Bronchodilator response (%)**	Treatment			
											
											BDP (μg/day)	Theophylline (mg/day)	Clenbuterol (μg/day)	Carbocysteine (mg/day)
1	54	M	161	Int	Moderate	420	-	-	2.5	15.2	800	400	40	0
2	72	F	147	Ext	Moderate	642	Mite, HD	AR	0.31	31.5	800	400	0	0
3	70	M	161	Ext	Mild	312	Mite, HD, Cedar	-	0.08	20.2	0	600	0	0
4	71	F	140	Int	Mild	17	-	-	1.25	17.6	800	0	0	0
5	83	M	154	Ext	Moderate	345	Mite, HD, Cedar	-	5	17.1	800	400	40	1500
6	71	M	165	Ext	Moderate	146	Mite, HD	AR	1.25	15.6	0	0	40	0
7	77	F	144	Int	Mild	51	-	-	0.31	17.9	0	0	0	1500
8	71	M	155	Int	Mild	42	-	-	2.5	29.4	800	0	0	1500
9	80	M	152	Int	Moderate	66	-	-	1.25	39	800	0	0	0
10	75	M	162	Ext	Mild	143	Candida	-	2.5	14.1	800	0	0	0
11	80	F	145	Ext	Mild	3	HD, Cedar	-	0.08	37.1	800	0	0	0
12	63	F	154	Ext	Moderate	77	Cedar	AR	1.25	14.7	800	0	0	0
13	77	F	142	Int	Mild	105	-	-	5	17	0	400	20	0
14	70	M	155	Int	Moderate	82	-	-	0.31	15.4	800	0	0	1500
15	70	F	151	Ext	Mild	467	Mite, HD	-	2.5	20.4	800	400	40	0
16	72	F	150	Int	Mild	57	-	-	5	22.3	600	0	0	1500
17	81	M	163	Int	Moderate	64	-	-	0.31	33.4	800	600	40	1500
18	71	M	150	Int	Moderate	107	-	-	5	16.4	800	400	40	0
19	80	M	160	Int	Mild	87	-	-	2.5	29.5	0	400	0	0
20	68	M	167	Ext	Mild	264	Cedar	-	5	27	0	400	40	0
21	80	F	152	Int	Mild	54	-	-	2.5	17.3	0	400	0	0

Ext, extrinsic; Int. Intrinsic; HD, house dust; AR, allergic rhinitis; UR, urticaria; BDP, beclomethasone diproprionate inhalation.

* PC20-FEV1 shows concentration of inhaled methacholine causing a 20% fall in FEV1.

** Bronchodilator response means percent increase in forced expiratory volume in 1s (FEV1) from the baseline value after inhalation of 300 μg of salbutamol sulfate.

All patients used inhaled β2-agonists (salbutamol or procaterol) on demand.

Each asthmatic patient satisfied the American Thoracic Society definition of asthma, with symptoms of episodic wheezing, cough, and shortness of breath responding to bronchodilators, and reversible airflow obstruction documented on at least one previous pulmonary function study [6]. Reversibility was defined as greater than 12 % and 200 ml increase in the forced expiratory volume in one second (FEV1) following a bronchodilator inhalation (Table 1). All patients had bronchial hyperresponsiveness as shown in Table 1. Classification of asthma severity was defined according to Global Strategy for Asthma Management and Prevention. Patients with atopy were recognized as having a hereditary tendency to produce IgE antibodies against common environmental allergens [7]. This study was carried out when symptoms were mild and stable, while patients were taking oral theophylline, oral (short-acting clenbuterol) and/or aerosol β2-agonists (short-acting procaterol), inhaled steroids (beclomethasone dipropionate), inhaled anti-cholinergic agents (oxitropium bromide) and/or mucolytic agents (carbocysteine) according to previous reports [8-10]. They had not received oral steroids for at least eight weeks.

### Assessment of cough reflex sensitivity to inhaled capsaicin

Cough reflex sensitivity was assessed by capsaicin provocation test [11]. Capsaicin (30.5 mg) was dissolved in Tween 80 (1 mL) and ethanol (1 mL) and then dissolved in physiological saline (8 mL) to make a stock solution of 1 × 10^-2 ^M, which was stored at -20°C. This solution was diluted with physiological saline to make solutions starting at a concentration of 0.49 μM and increasing it by doubling concentrations up to 1000 μM. Each subject inhaled a control solution of physiological saline followed by progressively increasing concentrations of the capsaicin solution. Solutions were inhaled for 15 s every 60 s, by tidal mouth-breathing wearing a noseclip from a Bennett Twin nebulizer (3012-60 cc, Puritan-Bennett Co., Carlsbad, California, USA). Increasing concentrations were inhaled until five or more coughs were elicited. The nebulizer output was 0.21 mL/min. The number of capsaicin-induced coughs was counted by a blinded medical technician in our pulmonary function laboratory. The cough threshold was defined as the lowest concentration of capsaicin that elicited five or more coughs.

### Study protocol (Figure 1)

**Figure 1:**
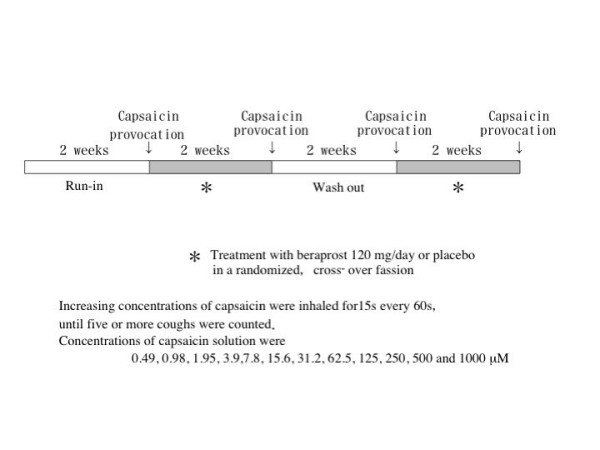
Study protocol.

The medication was stopped at 9.00 p.m. on the previous day to allow a washout time of 12 h or more before the measurement of cough threshold to inhaled capsaicin at 10.00 a.m. on each test day to reduce the diurnal variability of the cough response.

Each patient attended 4 times separated by 2 weeks, at the same time each day. Control measurement of capsaicin cough threshold was carried out 2 weeks before initiation of the first treatment (run-in). Two weeks treatment with beraprost sodium or placebo was performed separated by a two-week washout period in a randomized, cross-over fashion. Two beraprost sodium tablets (40 μg) and their placebo were taken orally three times a day for 14 days and at 8.00 a.m. on the test day. FEV1 was measured on a dry wedge spirometer (Transfer Test, P.K. Morgan Ltd., UK) before capsaicin challenge to assess the bronchoactive effect of the treatment regimens. Serum total IgE levels and the number of peripheral eosinophils were measured to assess anti-allergic effect of the test drugs.

### Data analysis

Capsaicin cough threshold values were expressed as geometric mean with geometric standard error of the mean (GSEM). Forced vital capacity (FVC), FEV1 and maximal mid expiratory flow (MMF) were shown as arithmetic mean values ± SEM. The FVC values, the FEV1 values and the MMF values were compared between each pair of the four groups (run-in, washout, beraprost sodium and placebo) by the Wilcoxon signed-ranks test. A p-value of 0.05 or less was taken as significant.

## Results

**Figure 2:**
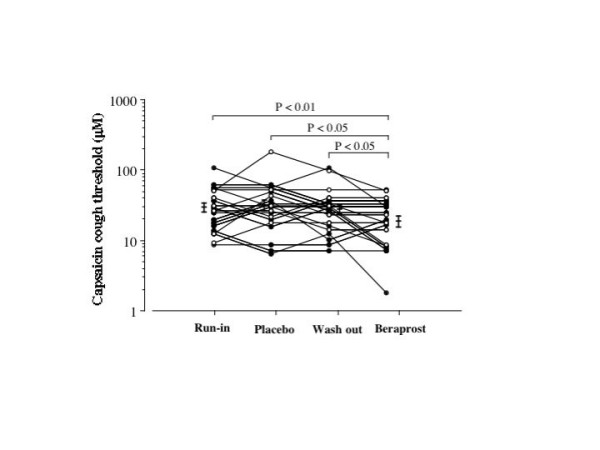
Individual data of capsaicin cough threshold at run-in period, at washout period and on treatment with beraprost and placebo in patients with stable bronchial asthma. Each horizontal bar represents geometric mean value. Closed circles and open circles represent patients undergoing steroid inhalation therapy and patients without steroid inhalation therapy, respectively. P values: Wilcoxon signed-ranks test using logarithmically transformed values.

Cough threshold to inhaled capsaicin before each treatment (run-in, washout) and after treatment with beraprost and placebo are shown in Figure 2. Geometric mean values for the cough threshold were 29.5 (GSEM 1.17) μM in the run-in period, 26.5 (GSEM 1.18) μM in the washout period, 17.8 (GSEM 1.20) μM after beraprost treatment and 30.3 (GSEM 1.21) μM after placebo treatment. The cough threshold after the beraprost treatment was significantly (p < 0.05) lower than the value after the placebo treatment. FVC, FEV1 or MMF value was not significantly different between run-in period, washout period, beraprost treatment and placebo treatment as shown in Table 2.

**Table 2:** Pulmonary function on beraprost and placebo treatments in patients with bronchial asthma

	Run-in	Placebo	Washout	Beraprost
FVC as % pred. (%)	96.8 ± 5.7	103.4 ± 3.3	104.4 ± 3.1	103.4 ± 3.4
FEV1 as% pred. (%)	90.9 ± 5.7	94.1 ± 5.5	93.0 ± 5.6	93.2 ± 5.6
MMF as% pred. (%)	50.7 ± 6.7	52.0 ± 6.0	50.1 ± 6.4	51.5 ± 6.4

Data are shown as standard error of the mean for FVC, FEV_1 _and MMF.

* p < 0.05 compared with each control value (Wilcoxon signed-ranks test).

**Figure 3:**
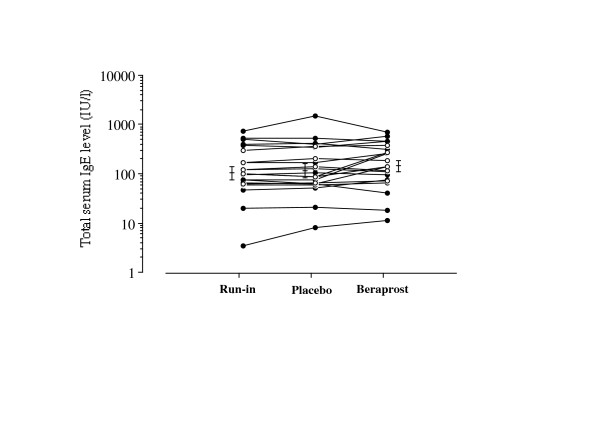
Individual data of serum IgE at run-in period, at washout period and on treatment with beraprost and placebo in patients with stable bronchial asthma. Each horizontal bar represents geometric mean value. Closed circles and open circles represent patients undergoing steroid inhalation therapy and patients without steroid inhalation therapy, respectively. P values: Wilcoxon signed-ranks test using logarithmically transformed values.

**Figure 4:**
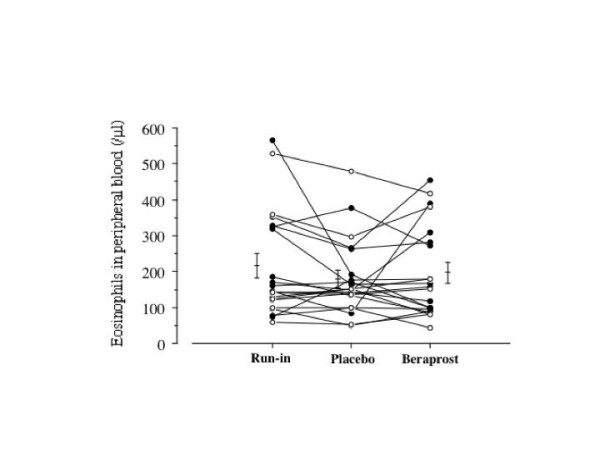
Individual data of peripheral blood eosinophils at run-in period, at washout period and on treatment with beraprost and placebo in patients with stable bronchial asthma. Each horizontal bar represents geometric mean value. P values: Wilcoxon signed-ranks test.

Figure 3 and figure 4 show the changes in serum IgE and peripheral blood eosinophils, respectively. Treatment with beraprost did not affect the IgE production or peripheral blood eosinophil count.

## Discussion

The present study showed that two-week treatment with a stable PGI2 analogue, beraprost, decreased the cough threshold to inhaled capsaicin in asthmatic patients. No difference could be found in the baseline pulmonary function, IgE production or peripheral eosinophil count between beraprost and placebo treatments. These findings suggest that PGI2 enhances the cough reflex sensitivity in the asthmatic airway.

Cough is one of the main symptoms of bronchial asthma which can profoundly and adversely affect the quality of patient's lives and social activities, whereas the mechanisms underlying the cough remain obscure. Previous researchers [12] indicated that cough receptors are stimulated by local bronchoconstriction. This finding may be one of the causes of cough in bronchial asthma. However, recent studies about cough variant asthma (CVA) revealed normal baseline pulmonary function and mild bronchial hyperresponsiveness [13,14]. Our previous study has also demonstrated that inhaled procaterol in a dose sufficient to produce bronchodilation has no effect on airway cough receptor sensitivity in asthma [15]. O'Connell and colleagues have reported that cough reflex sensitivity is increased in some asthmatic patients suffering from daily coughing and recovers to normal range after relief of the cough on treatment [16]. These findings suggest that cough reflex hypersensitivity is another mechanism of chronic non-productive cough in asthma, in addition to cough receptor stimulation by local bronchoconstriction [12].

It has been revealed that inflammatory mediators such as arachidonate metabolites play major roles in the pathogenesis of bronchial asthma, however, the relationship between inflammatory mediators and airway cough reflex sensitivity remains obscure. Some studies indicated that some inflammatory mediators might modulate the sensitivity of cough reflex [1,17]. We showed that intrinsic thromboxane A2 (TxA2) is a possible modulator augmenting both airway cough reflex sensitivity and bronchial responsiveness while it does not have bronchoconstricting effect in stable asthmatics [1,18,19]. Other researchers reported that prostaglandin F2α (PGF2α) enhances airway cough reflex sensitivity with bronchoconstricting effect [2,20]. It has been also shown that inhaled prostaglandin E2 (PGE2), which acts as a bronchodilator, enhances cough reflex sensitivity [20,21]. Although cysteinyl leukotrienes (cLTs) play an important role in bronchomotor tone of the asthmatic airway, their role in cough reflex sensitivity is controversial [19,22]. These findings indicate that arachidonate metabolites including prostaglandins may have variable roles in the local control of the cough reflex with no relation to bronchoconstriction.

It has been known that PGI2 is the most abundant prostanoid generated on IgE-dependent challenge of human lung tissue in vitro [2,3]. Others reported that alveolar macrophages are able to synthesize a large amount of PGI2 [4]. These findings imply that PGI2 plays some role in asthmatic airway. Although PGI2 causes relaxation of isolated precontracted human bronchus [23], its clinical effect is limited: short-term protection against immediate bronchoconstriction provoked by exercise [24], and nebulized distilled water [24] but not by allergen [25] or aspirin [26]. Therefore, the exact role of PGI2 in asthmatic airway remains obscure. Hardy et al. reported an irritative effect of single inhalation of PGI2 on human airways [27], but influence of repeated administration has not been studied. Szczeklik and their colleagues also reported that four out of twelve asthmatic patients complained of coughing during PGI2 inhalation [28]. However, these previous reports have not investigated the change of cough reflex sensitivity. Thus the exact role of PGI2 in airway cough reflex sensitivity also remains unknown. We observed that some patients complained of coughing on treatment with beraprost but none did with placebo. The implication of this study is that PGI2 may be involved in the pathogenesis of cough reflex sensitivity rather than bronchodilation and it may explain the role of PGI2 in the asthmatic airway which has been unknown so far.

Overall, our data support the conclusion that inhibition of PGI2 formation or action may be a novel treatment for chronic non-productive cough in asthmatic airway, especially in cough variant asthma or cough predominant asthma with normal baseline pulmonary function. This is the first report demonstrating the role of PGI2 in cough reflex sensitivity in the asthmatic airway. Further studies may be required to elucidate the role of PGI2 in other eosinophilic bronchial disorders presenting with non-productive cough with normal baseline pulmonary functions [29-31].

## Abbreviations

cLT = cysteinyl leukotriene; CVA = cough variant asthma; FEV1 = forced expiratory volume in one second; FVC = forced vital capacity; GSEM = geometric standard error of the mean; PGE2 = prostaglandin E2; PGF2α = prostaglandin F2α; PGI2 = prostaglandin I2; MMF = maximal mid expiratory flow; TxA2 = thromboxane A2.
